# First-in-human, double-blind, randomized phase 1b study of peptide immunotherapy IMCY-0098 in new-onset type 1 diabetes: an exploratory analysis of immune biomarkers

**DOI:** 10.1186/s12916-024-03476-y

**Published:** 2024-06-21

**Authors:** Jean Van Rampelbergh, Peter Achenbach, Richard David Leslie, Martin Kindermans, Frédéric Parmentier, Vincent Carlier, Nicolas Bovy, Luc Vanderelst, Marcelle Van Mechelen, Pierre Vandepapelière, Christian Boitard

**Affiliations:** 1 Imcyse S.A, Avenue Pré-Aily 14, Liège, 4031, Belgium; 2 Institute of Diabetes Research, Helmholtz Zentrum München, German Research Center for Environmental Health, Munich-Neuherberg, Germany; 3 Forschergruppe Diabetes, Technical University Munich, Klinikum Rechts Der Isar, Munich, Germany; 4 Department of Immunobiology, Queen Mary University of London, London, UK; 5 Ariana Pharmaceuticals SA, Paris, France; 6 Inserm U1016, Cochin Institute, Paris, France; 7 Medical Faculty, Université de Paris, Paris, France

**Keywords:** Type 1 diabetes, Immunotherapy, T cells, Beta cells, Exploratory analysis, Immune biomarker machine learning

## Abstract

**Background:**

IMCY-0098, a synthetic peptide developed to halt disease progression via elimination of key immune cells in the autoimmune cascade, has shown a promising safety profile for the treatment of type 1 diabetes (T1D) in a recent phase 1b trial. This exploratory analysis of data from that trial aimed to identify the patient biomarkers at baseline associated with a positive response to treatment and examined the associations between immune response parameters and clinical efficacy endpoints (as surrogates for mechanism of action endpoints) using an artificial intelligence-based approach of unsupervised explainable machine learning.

**Methods:**

We conducted an exploratory analysis of data from a phase 1b, dose-escalation, randomized, placebo-controlled study of IMCY-0098 in patients with recent-onset T1D. Here, a panel of markers of T cell activation, memory T cells, and effector T cell response were analyzed via descriptive statistics. Artificial intelligence-based analyses of associations between all variables, including immune responses and clinical responses, were performed using the Knowledge Extraction and Management (KEM^®^) v 3.6.2 analytical platform.

**Results:**

The relationship between all available patient data was investigated using unsupervised machine learning implemented in the KEM^®^ environment. Of 15 associations found for the dose C group (450 μg subcutaneously followed by 3 × 225 μg subcutaneously), seven involved human leukocyte antigen (HLA) type, all of which identified improvement/absence of worsening of disease parameters in DR4^+^ patients and worsening/absence of improvement in DR4^−^ patients. This association with DR4^+^ and non-DR3 was confirmed using the endpoints normalized area under the curve C-peptide from mixed meal tolerance tests where presence of DR4 HLA haplotype was associated with an improvement in both endpoints. Exploratory immune analysis showed that IMCY-0098 dose B (150 μg subcutaneously followed by 3 × 75 μg subcutaneously) and dose C led to an increase in presumed/potentially protective antigen-specific cytolytic CD4^+^ T cells and a decrease in pathogenic CD8^+^ T cells, consistent with the expected mechanism of action of IMCY-0098. The analysis identified significant associations between immune and clinical responses to IMCY-0098.

**Conclusions:**

Promising preliminary efficacy results support the design of a phase 2 study of IMCY-0098 in patients with recent-onset T1D.

**Trial registration:**

ClinicalTrials.gov NCT03272269; EudraCT: 2016–003514-27.

**Supplementary Information:**

The online version contains supplementary material available at 10.1186/s12916-024-03476-y.

## Background

Type 1 diabetes (T1D) is a chronic autoimmune disease that accounts for approximately 5–10% of all diabetes diagnoses and > 85% of the diagnoses made in youth [1, 2]. The disease is characterized by loss of sensitivity to insulin-producing β cells, which leads to β cell destruction, a decline in endogenous insulin secretion and, consequently, hyperglycemia [3]. Therapies that aim to modify the underlying cause of disease, such as antigen or antibody immunotherapies, are of particular interest and have demonstrated efficacy and safety in previous studies [4–6]. IMCY-0098 is an Imotope™, a linear synthetic peptide encompassing a proinsulin C20-A1 epitope and a thioredox motif, which has been shown to induce a cytolytic phenotype in a human CD4 T cell line and is able to eliminate antigen-presenting cells (APCs) that present the proinsulin epitope (data not shown). Interestingly, treatment with a mouse insulin-derived Imotope™ offered protection from diabetes in a non-obese diabetes mouse model [7]. Furthermore, cytolytic CD4 cells also eliminate autoreactive pathogenic T cells upon cognate interaction with the same APCs, irrespective of the epitope presented [7, 8]. The IMCY-0098 peptide has shown good binding capacity to DR3 and DR4 polymorphisms, which are strongly associated with T1D [9, 10].

Machine learning is an important component of artificial intelligence that has been integrated into many fields, including drug discovery, drug development, and the prediction of drug efficacy [11]. The Knowledge Extraction and Management (KEM^®^) analytical platform is a machine learning system used to systematically extract unsupervised relationships between collected variables with no predefined hypothesis [12]. KEM^®^ artificial intelligence uses the formal concept analysis framework [13] that has been successfully applied in different domains, including drug discovery, studies for the identification of patient selection biomarkers for therapeutic responses and genomic characterization of complex diseases [14–18]. KEM^®^ systematically identifies all groups of patients with shared characteristics and generates corresponding association rules that can detect patterns and relationships in heterogeneous databases [18–22]. This approach (formal concept analysis) is different from predictive modeling as it allows for identification of the most relevant hypothesis that is consistent with the data [23]. Formal concept analyses are able to extrapolate significant relational detail from datasets with small sample size and a large number of variables [24], and association rules help to detect rare signals. Drawing conclusions from small sample sizes is of importance for medical research [25], and these methods may be useful for this purpose.

Data from a recent phase 1b trial (NCT03272269) demonstrated a promising safety profile of ICMY-0098 [10]. In this exploratory analysis of samples from this trial, we aimed to use an artificial intelligence-based approach, suitable for small patient numbers, of unsupervised explainable machine learning to explore the associations between all variables. Specifically, we aimed to identify the biomarkers at baseline associated with positive response to treatment and to examine the associations between immune response parameters and clinical efficacy endpoints as surrogate endpoints for mechanism of action. This study aimed to identify patient and disease characteristics associated with response to treatment with IMCY-0098 using the KEM^®^ analytical platform, to gain further insights into the mode of action of IMCY-0098, and to inform the design of future clinical studies.

## Methods

### Study design

This was an exploratory analysis of data from a phase 1b, dose-escalation, randomized, placebo-controlled study of IMCY-0098 in patients with recent-onset T1D (NCT03272269). The study design was as described previously [10]. Briefly, 41 patients diagnosed with T1D ≤ 6 months before the start of the study were randomized 3:1 to receive IMCY-0098 or placebo. Patients allocated to receive IMCY-0098 were sequentially enrolled to receive dose A (50 μg subcutaneously followed by 3 × 25 μg subcutaneously), dose B (150 μg subcutaneously followed by 3 × 75 μg subcutaneously), or dose C (450 μg followed by 3 × 225 μg subcutaneously). Treatment was administered in four doses with aluminum hydroxide adjuvant (alum) at a concentration of 500 μg/mL in 2-week intervals from weeks 0 to 6 with follow-up at weeks 12, 18, and 24. The study included patients who were HLA DR3 + and/or DR4 + ; had ≥ 1 autoantibody against GAD65, IA-2, or ZnT8; and had fasting C-peptide at screening (week −4) > 0.2 nmol/L and/or stimulated C-peptide ≥ 0.4 nmol/L. Data were collected in a double-blind manner until all patients completed the study to week 24 or were prematurely withdrawn from the study.

### Study objectives and endpoints

The primary objective of the phase 1b study was to assess the safety of IMCY-0098 and the secondary objective was to assess the clinical response (previously published [10]). There was an additional exploratory objective to characterize the immune response to IMCY-0098 treatment using a panel of markers of T cell activation, memory T cells, and effector T cell response. As such, the immune endpoints described in the current study included detection of CD4^+^ T cells specific for epitope C20-A1 of proinsulin (peptide sequence included in IMCY-0098), impact on effector CD4^+^, and CD8^+^ T cell responses specific for insulin, glutamic acid decarboxylase 65 (GAD65), and islet-specific glucose-6-phosphatase catalytic subunit-related protein (IGRP).

The main time point for all endpoints was 24 weeks; however, other time points were also considered in the analysis.

### Study procedures and assessments

Exploratory immunologic assessments were performed in a specialized and independent laboratory. Approximately 100 mL of blood was collected at each time point (weeks −4, 0, 6, 12, and 24) for peripheral blood mononuclear cells (PBMC) preparation. All immunoassay analyses were performed using fluorescence-activated cell sorting techniques.

### Clinical response

A clinical response parameter, expected area under the curve (AUC) C-peptide, was derived based on the difference between the normalized AUC measured during mixed meal tolerance tests (MMTT tests) and the AUC expected values given the general disease evolution, as described previously [26]. The AUC expected values used in this study were chosen based on the time to diagnosis (< 12 months for all 41 patients): −0.0245 pmol/mL/month (95% confidence interval: −0.0271, −0.0215).

### Immune response

**Fig. 1:**
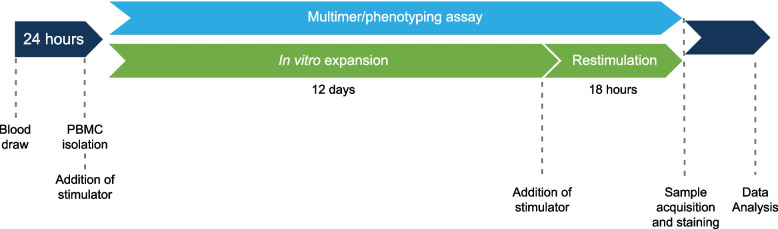
Summary of immune response methodology

PBMCs were isolated from blood using Ficoll density gradient centrifugation and analyzed within 24 h of blood collection or after in vitro stimulation that aimed to expand specific T cells with a cocktail of all peptides (Peptivator^®^ human insulin, Miltenyi, Bergisch Gladbach, Germany), GAD65 (Peptivator^®^ human GAD65, Miltenyi, Bergisch Gladbach, Germany), three IGRP major epitopes (Lifetein, Somerset, NJ, USA), or the C20-A1 epitope of proinsulin included in IMCY-0098. Negative and positive controls were of peptide diluent or *Staphylococcal* enterotoxin B, respectively. Freshly isolated or in vitro expanded PBMCs were stimulated with indicated antigens (using a 12-day expansion phase with restimulation 18 h before staining) for subtype markers (CD3, CD4, CD8), naïve/memory T cell differentiation markers (CCR7, CD45RA), and proliferation marker (antigen Kiel 67 [Ki67]) and function (interleukin [IL]-17, interferon gamma [IFN-γ], lysosomal-associated membrane protein-1 [CD107a], granzyme B and perforin), before flow cytometry analysis on a BD^®^ LSR cytometer (Becton Dickinson, Erembodegem, Belgium). A summary of the assay methodology is presented in Fig. 1.

For detection of specific CD8^+^ and CD4^+^ T cells after the in vitro expansion phase, HLA-A*02 pentamers loaded with Preproinsulin, IGRP, or GAD65 epitopes; DRB1*03:01 tetramer loaded with PI C20-A1 peptide or GAD65 peptide; and DRB1*04:01 tetramer loaded with K to S substitution in position 9 of PI C20-A1 peptide [27, 28] or GAD65 peptide were combined with multiparameter flow cytometry. T cell subsets were analyzed using specific markers: naïve (CD45RA + CCR7 +), central memory (CD45RA-CCR7 +), effector memory (CD45RA-CCR7–), and terminal effector (CD45RA + CCR7–).

### Delta ratios

For both clinical and immune endpoints that were measured at different timepoints, delta ratios were calculated. Firstly, delta ratios were calculated using the following formula to measure the evolution of the disease from baseline, where *Vn* = visit and *V*baseline = baseline visit:

$$\Delta{endpoint} \left(V{baseline} \to Vn\right)=\frac{\,{endpoint} \left(Vn\right)- {endpoint}\,\left(V{baseline}\right)}{{endpoint} \,(V{baseline})}$$

*Vn* = visit n; *V*baseline = baseline visit (randomization).

Secondly, all numerical parameters (delta ratios and baseline characteristics) were discretized as described here: (1) delta ratios were discretized in three categories (increasing [delta > 0], stable [delta = 0], or decreasing [delta < 0]), and (2) baseline characteristics were categorized using an equal-frequency tertile binning as high (top tertile), medium (middle tertile), and low (bottom tertile).

### Statistical analysis

The study sample size for this phase 1 study is not based on any statistical hypothesis testing, as is often the case for first-in-human studies where there is little information available beforehand. However, it was aligned with a similar study of peptide immunotherapy [29] and determined as adequate to support preliminary assessment of safety, efficacy, and immune response. This size favored a higher patient number in the dose C group as this higher dose was expected to achieve greater immunogenicity versus a lower-dose group, an important consideration for the current analysis [10]. This approach was fully endorsed by seven regulatory authorities.

### Formal concept analysis-based artificial intelligence

Artificial intelligence-based association rule analyses between immune responses and clinical responses were exploratory in nature; the analysis was included in the study protocol/statistical analysis plan, but no detailed method was pre-specified.

**Fig. 2:**
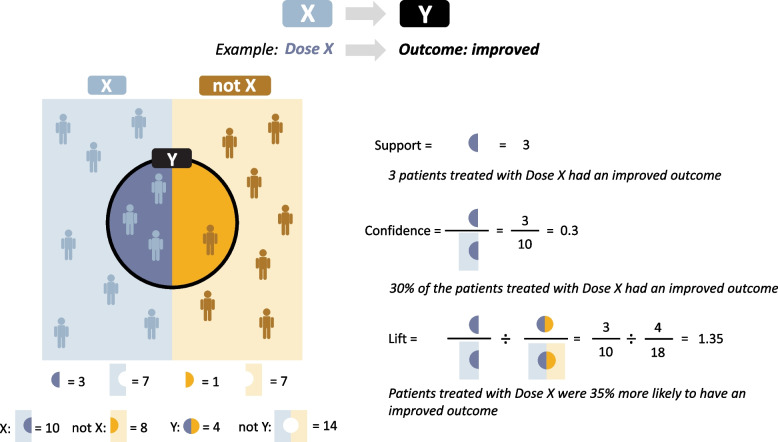
Example application of quality measures support, confidence, and lift to association rules

The KEM^®^ v 3.6.2 analytical platform is an explainable AI platform that systematically implements unsupervised mining of association rules and was used to systematically extract unsupervised relations between all variables collected with no predefined hypothesis [12, 19, 30]. An association rule is defined as a relationship *X* → *Y*, where *X* (left part) and *Y* (right part) can be a unique descriptor or combination of descriptors (Fig. 2). Five quality measures were used to characterize and rank all generated rules: support (the number of patients for whom the association was identified), confidence (the probability of having *Y* within patients verifying *X*), lift (the ratio between confidence and the probability of having *Y* in the whole dataset; lift describes the enrichment brought by considering patients verifying *X* instead of all patients), and two *p*-values (derived from Fisher’s exact test and Mann–Whitney–Wilcoxon). Statistical significance was neither necessary nor sufficient to indicate a causal relationship. Multiple tests were not taken into account in the rule-of-association approach; formal concept analysis methods generate relationships between descriptors in an exhaustive manner despite large combinatorial space and compute metric values for each relationship, so multiple analyses/corrections are not necessary.

Association between clinical outcomes at visit 8 (week 24), dose, and the evolution of immune markers was explored by focusing on the subset of relations where dose was necessary and sufficient. In addition, relations between all available descriptors were systematically characterized, and the associations between treatment groups, clinical outcomes (combination of antecedents), and immune response (consequent) were analyzed using the KEM^®^ platform (Additional File 1: Fig. S1).

Post hoc analyses were performed to assess the association with DR4^+^ and non-DR3 using the following endpoints: normalized AUC C-peptide from MMTT, fasting C-peptide/glucose and insulin. Given the small study sample size and the HLA imbalance between groups, these analyses were performed by grouping the placebo group with dose A (untreated group) and dose B with dose C (treated group). An additional responder analysis was performed using the published quantitative response (QR) methodology adjusted based on data from the 6-month follow-up [31]. For the normalized AUC C-peptide, this model allows the calculation of an expected C-peptide value after a period based on baseline AUC and age at T1D onset. The observed value minus the model-based expected C-peptide value (QR) was defined to reflect the effect of the therapy.

## Results

Patient HLA haplotypes are presented in Additional File 1: Table S1. The most common HLA type was DR4^+^ only (46.3%), and most patients were HLA-A2:01 (63.4%).

### Clinical response

**Fig. 3:**
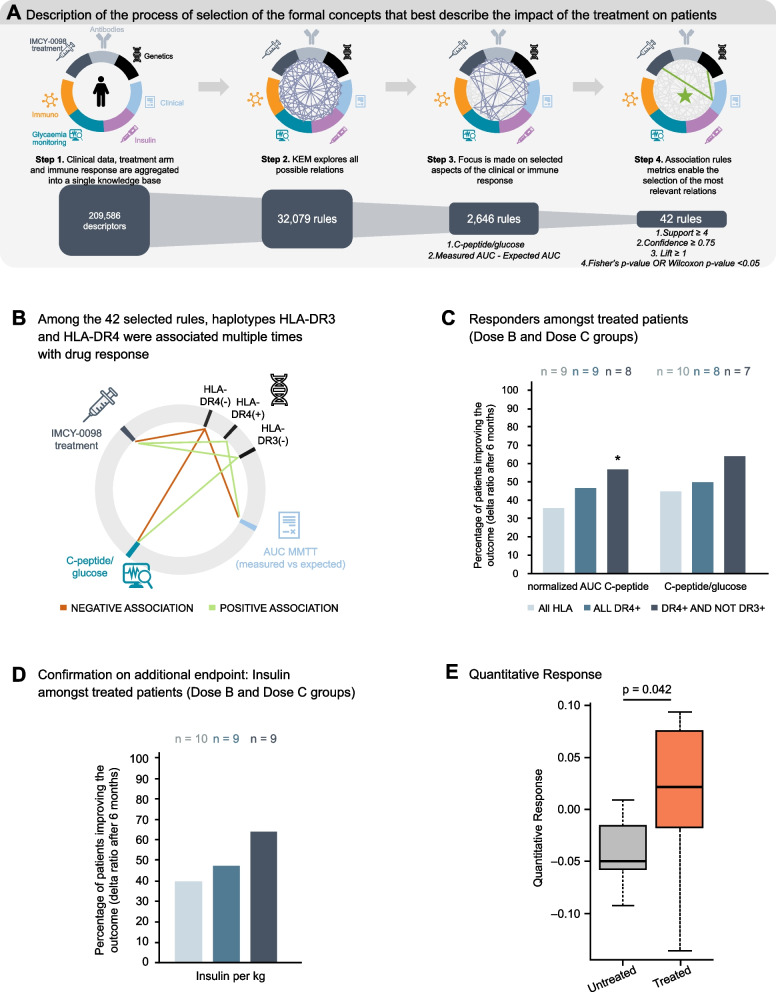
Formal concept analysis of associations between treatment group, clinical endpoints, immune response data, and subgroups. **A** Analysis workflow. Among all 32,079 generated rules, 12,638 relevant rules were explored to find associations for clinical response. Associations were selected for support ≥ 4, lift ≥ 1.25, confidence ≥ 0.75, and *p* ≤ 0.05. **B** Statistically significant associations involving treatment with IMCY-0098 dose C, improvement of clinical endpoints, and HLA type. **C**, **D** HLA-dependent changes in subjects among the dose B and dose C groups for **C** normalized AUC C-peptide from MMTT and fasting C-peptide/glucose and **D** insulin dose per kg. **E** Box plot of quantitative response for AUC C-peptide in untreated vs treated groups. Central lines represent median values, boxes represent interquartile range, and whiskers represent upper and lower 1.5 × interquartile range, respectively. Dose B: 150 μg at week 0 followed by 3 × 75 μg; dose C: 450 μg at week 0 followed by 3 × 225 μg. * Fischer’s *p* = 0.044. AUC, area under the curve; HLA, human leukocyte antigen; MMTT, mixed meal tolerance test

KEM^®^ was used to systematically generate all association rules between the descriptors of the dataset (treatment dose and candidate subgroups, defined based on patient characteristics) and the endpoints (clinical or immune response at any visit) (Fig. 3A).

The analysis was focused on two endpoints selected for general knowledge, signal strength, and interpretability: fasting C-peptide/glucose ratio and the difference between the measured and the expected normalized AUC for C-peptide from MMTT. After applying the filters, 42 associations involving 18 clinical markers were selected (Additional File 1: Table S2). Of 15 associations found for the IMCY-0098 dose C treatment group, seven involved HLA type. All of the associations identified improvement/absence of worsening of disease parameters in DR4^+^ or DR3^−^ patients (two associations at two different visits) and worsening/absence of improvement in DR4^−^ patients (two associations at Visit 6) (Fig. 3B).

The proportion of responders among the treated group increases based on HLA DR4 positivity and absence of DR3. When focusing on the evolution of normalized AUC C-peptide from MMTT in DR3^−^ patients, this proportion is significantly higher compared with the untreated group: Fisher’s *p* = 0.044 (Fig. 3C). The grouping used for the analyses—the treated (i.e., dose B + C) and untreated (i.e., placebo + dose A) groups—was supported by the absence of any association rules regarding the HLA status of patients in the placebo and dose A groups, while the dose B and C groups shared common associations from the KEM analysis.

The postulated association with DR4 + and non-DR3 was confirmed using the insulin endpoint; the presence of DR4 HLA type and the absence of DR3 HLA type were associated with improvement of this endpoint in the post hoc analysis (Fig. 3D). In another *post hoc* analysis we looked at the normalized AUC C-peptide using the QR model approach. Briefly, this model allows calculation of an expected C-peptide value after a period based on baseline AUC and age at T1D onset. The observed minus the model-based expected C-peptide value (QR) is defined to reflect the effect of the therapy. The results showed that the quantitative response in the treated group was significantly higher versus the untreated group (Fig. 3E).

### Immune component analysis

**Fig. 4:**
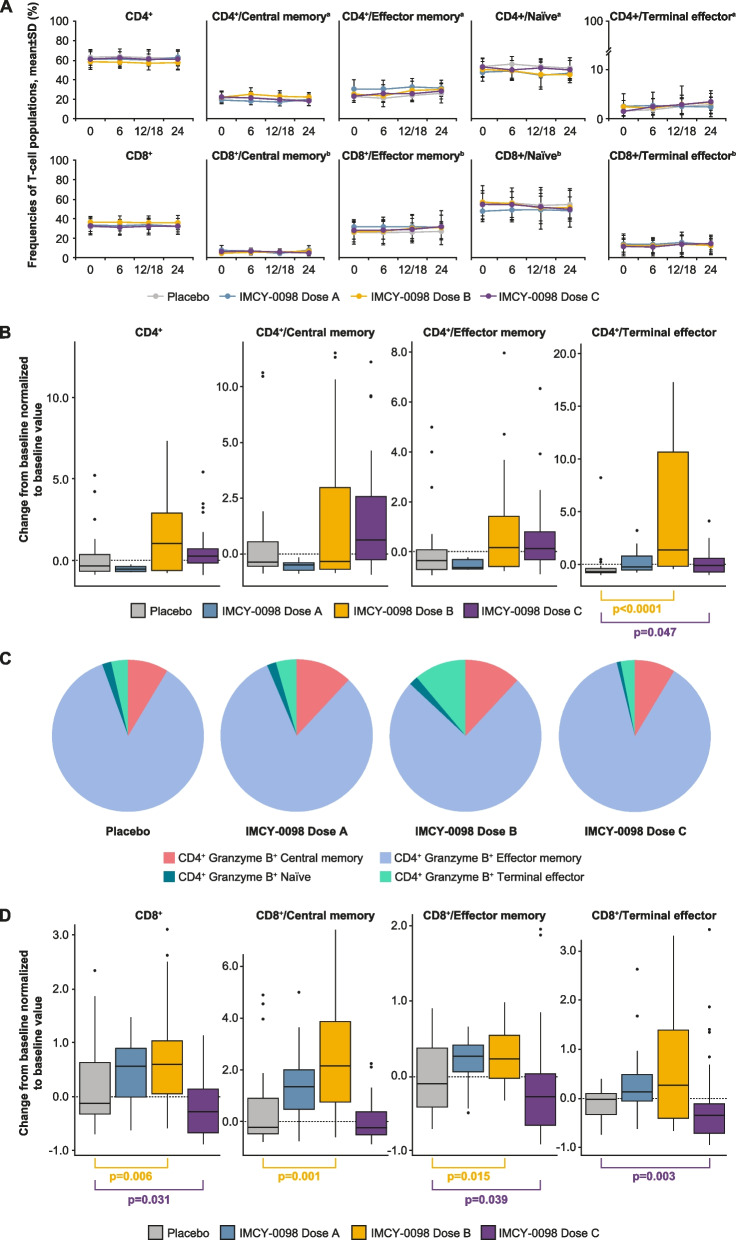
Immune response to IMCY-0098 treatment—intent-to-treat population. **A** Frequencies of different T cell subsets over time: naïve (CD45RA + CCR7 +), central memory (CD45RA-CCR7 +), effector memory (CD45RA-CCR7-), and terminal effector (CD45RA + CCR7 − ; no in vitro stimulation; error bars represent SD). **B** Change from baseline to week 24 in treatment-specific granzyme B^+^ cells within CD4 + T cell subsets after in vitro stimulation with IMCY0163 (insulin C20-A1). **C** Distribution of CD4^+^ granzyme B + T cells at week 24. **D** Change from baseline to week 24 in disease-specific Perforin + CD8^+^ T cells after in vitro stimulation with GAD65 and IGRP peptides. ^a^Frequency within total CD4^+^ cell population. ^b^Frequency within total CD8^+^ cell population. Box and whisker plots (**B**, **D**) show change of cell numbers from baseline to week 24, normalized to baseline value. Central lines represent median values, boxes represent interquartile range, and whiskers represent upper and lower 1.5 × interquartile range, respectively. *p*-values were obtained using Wilcoxon-Mann–Whitney test; *p* > 0.05 unless indicated otherwise. Dose A: 50 μg at week 0 followed by 3 × 25 μg; dose B: 150 μg at week 0 followed by 3 × 75 μg; dose C: 450 μg at week 0 followed by 3 × 225 μg. SD, standard deviation

Immune cells were analyzed using multiparameter multicolor flow cytometry for markers of T cell subtype, activation, proliferation, and function (Additional File 1: Table S3). Immune parameters following IMCY-0098 treatment and placebo were compared using three readouts: prevalence of general T cell subsets (to determine any unspecific treatment effect), induction of treatment (IMCY-0098)-specific cytolytic CD4^+^ T cells, and any changes in pathogenic T cells specific for β cell autoantigens. Across treatment groups, minor variations in the frequency of different T cell populations were observed during the study period. The numbers were similar for patients receiving IMCY-0098 and those receiving placebo, indicating that treatment with IMCY-0098 did not promote non-specific expansion or deletion of T cell subsets (Fig. 4A). These data support that the mode of action of IMCY-0098 is antigen-specific and targeted.

The change from baseline to week 24 in the number of granzyme B^+^ CD4^+^ cytolytic T cells in response to in vitro stimulation with the proinsulin epitope C20-A1 showed an upward trend in samples from IMCY-0098-treated groups (dose B and C) compared with placebo (Fig. 4B). A statistically significant difference was observed for IMCY-0098 dose B and dose C vs placebo for the change in CD4^+^ terminal effector cells (*p* < 0.001 and *p* = 0.047, respectively); differences did not reach statistical significance for central memory or effector memory CD4^+^ cells or for CD4^+^ cells overall (Fig. 4B). Importantly, central memory and effector memory cells were present across treatment groups at week 24 included, indicating that the cytolytic CD4^+^ T cell response may be sustained long term (Fig. 4C).

The number of Perforin^+^ CD8^+^ pathogenic T cells specific for β cell autoantigens measured following in vitro stimulation with GAD65 and IGRP showed varying trends with different doses of IMCY-0098 treatment (Fig. 4D).

### Immune response: unsupervised formal concept analysis

**Fig. 5:**
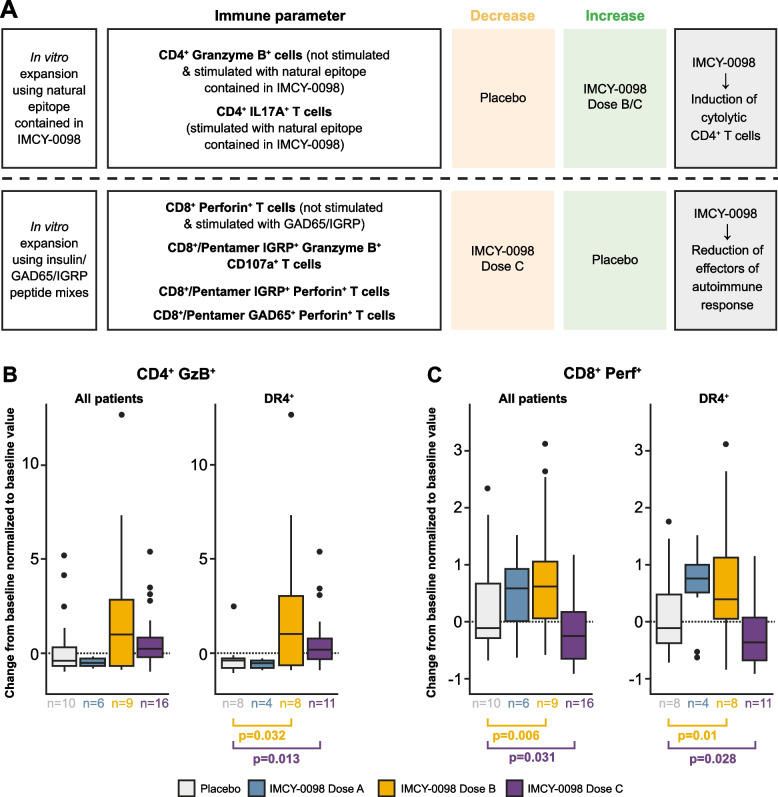
Immune response to IMCY-0098 treatment. Summary of immune parameters identified during formal concept analysis as associated with treatment (**A**) and changes in treatment-specific granzyme B^+^ CD4^+^ T cells (**B**) and disease-specific Perforin^+^ CD8^+^ T cells (**C**) after in vitro stimulation in all patients versus DR4 subgroup (see also Fig. 3 and Additional File 1: Fig. S2). Box and whisker plots (**B**, **C**) show change of cell numbers from baseline to week 24, normalized to baseline values. Central lines represent median values, boxes represent interquartile range, and whiskers represent upper and lower 1.5 × interquartile range, respectively. *p*-values were obtained using Wilcoxon-Mann–Whitney test; *p* > 0.05 unless indicated otherwise. Disease-specific CD8^+^ T cells refers to all T cells specific to any the disease-peptide loaded multimers. Dose A: 50 μg at week 0 followed by 3 × 25 μg; dose B: 150 μg at week 0 followed by 3 × 75 μg; dose C: 450 μg at week 0 followed by 3 × 225 μg

Several statistically significant associations were identified between immune response parameters and IMCY-0098 treatment (Fig. 5A, Fig. S2). Treatment with IMCY-0098 dose B and C showed a positive association with increases in unstimulated granzyme B^+^ CD4^+^ T cells as well as in granzyme B^+^ CD4^+^ and IL-17^+^ CD4^+^ T cells that were treatment-specific (responding to in vitro stimulation with proinsulin epitope C20-A1 that is contained in the IMCY-0098 sequence). Treatment with IMCY-0098 dose B showed an increase in Perforin^+^ CD8^+^ pathogenic T cells that responded to in vitro stimulation with GAD65/IGRP peptides, whereas dose C showed a reduction (Fig. 5B and C); this latter association met the significance criteria of the algorithm. Taken together, these results suggest that IMCY-0098 dose C modulates immune response in T1D by promoting the generation of treatment-specific cytolytic CD4^+^ T cells and inhibiting pathogenic autoreactive CD8^+^ T cells.

The change from baseline to week 24 in granzyme B^+^ CD4^+^ cytolytic T cells expanded in vitro using proinsulin epitope C20-A1 was significantly higher in DR4^+^ patients treated with IMCY-0098 dose B or dose C vs placebo (*p* = 0.032 and *p* = 0.013, respectively); statistical significance was not reached for the overall patient population (Fig. 5B). The change from baseline to week 24 in Perforin^+^ CD8^+^ pathogenic T cells expanded in vitro using GAD65/IGRP peptides was significantly higher in patients treated with IMCY-0098 dose B vs placebo (*p* = 0.006 and *p* = 0.01 for all patients and for DR4^+^ subgroup, respectively; Fig. 5C). This change was significantly lower in patients treated with IMCY-0098 dose C vs placebo (*p* = 0.031 and *p* = 0.028 for all patients and for DR4^+^ subgroup, respectively; Fig. 5C). Reduction of pathogenic CD8^+^ T cells in response to IMCY-0098 dose C treatment was noted for several other HLA type subgroups: DR3^−^, DR3/DR4, DR4X (data not shown).

### Association between clinical and immune response identified using formal concept analysis

**Fig. 6:**
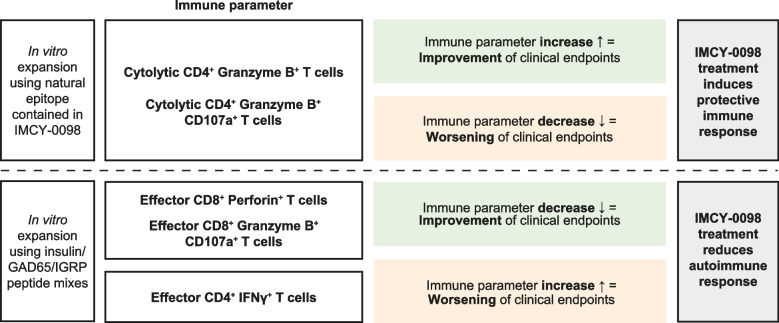
Association between clinical outcomes and immune response markers. Results are shown for week 24 for patients receiving IMCY-0098 dose B or C. Associations were selected for confidence ≥ 0.75, support ≥ 4, and *p* ≤ 0.05. Data for IMCY-0098 dose B and dose C treatment groups were pooled for this analysis. Dose B: 150 μg at week 0 followed by 3 × 75 μg; dose C: 450 μg at week 0 followed by 3 × 225 μg. GAD, glutamic acid decarboxylase; IGRP, islet-specific glucose-6-phosphatase catalytic subunit-related protein; IFN, interferon

Improvement and worsening of several clinical endpoints showed significant associations with increases and decreases of immune cell markers in the dose B and C groups (Fig. 6; Fig. S3). For example, in treatment groups IMCY-0098 dose B and dose C, decrease in insulin use showed a positive association with the increase in granzyme B^+^ CD4^+^ cytolytic T cells after in vitro expansion using insulin C20-A1 epitope (the natural epitope included in IMCY-0098: Fig. S4). In addition, increase of daily insulin dose was significantly associated with an increase in CD8^+^ pathogenic T cells after in vitro expansion with peptides derived from insulin, GAD65, or IGRP (data not shown). Similar trends were observed for associations between increase in various subpopulations of CD8^+^ pathogenic T cells and worsening of other clinical endpoints, such as fasting C-peptide and glucose levels and frequency of hypoglycemic events (data not shown). Conversely, in the placebo group, only very few non-relevant associations could be identified (Fig. S3).

Together, these results suggest that clinical improvement in patients treated with IMCY-0098 was associated with an increase in protective immune response (cytolytic T cells) and a reduction in autoimmune response (pathogenic T cells) (Fig. 6 and Additional File 1: Fig. S3).

## Discussion

A growing body of literature suggests the existence of different endotypes and data-driven analyses help identify responders to treatment in diabetes [26, 32–35]. Previous studies applying multivariable analysis to clinical outcomes in T1D showed that certain baseline characteristics of patients and their disease may be associated with long-term outcomes [26]. To overcome the limitation of small patient numbers and to obtain further insights into patient outcomes with IMCY-0098 treatment, our exploratory analysis included artificial intelligence-driven analysis of clinical and immune response using KEM^®^ [12, 19, 30, 36]. Although not specifically designed for the analysis of clinical trial data, rule extraction algorithms are common tools widely used in data mining in various domains. This returned a number of statistically significant associations between treatment and clinical outcomes and between immune response and clinical outcomes. In particular, we found multiple statistically significant associations between treatment with IMCY-0098, generation of potentially protective cytolytic CD4^+^ T cells, and inhibition of pathogenic autoreactive CD8^+^ T cells, suggesting that IMCY-0098 may elicit protective immune responses in humans.

Doses B and C showed similar increases in cytolytic CD4^+^ T cells, but only dose C significantly downregulated autoreactive CD8^+^ T cells. There are many possible reasons for this difference; phenotypic traits of cytolytic CD4^+^ T cells aside, lytic markers (such as granzyme B and CD107a) could differ slightly and thereby influence efficacy. Dose B was associated with an expansion of more terminally differentiated CD4^+^ T cells which suggests decreased effector capacity and weaker potency with which to control the immune response. This dosing effect should be evaluated in further studies with increased dosages and deeper phenotypic analysis of induced cytolytic CD4^+^ T cells by single cell analysis or cytometry by time of flight (CyTOF) technologies. It is also important to note that the impacted CD8^+^ T cells were of other specificities than treatment-induced CD4^+^ T cells, suggesting the action of a potent bystander killing mechanism.

Previous studies of genetic predisposition and recent studies assessing the benefit of immunotherapy for T1D suggest that patients’ HLA type could play a role in clinical efficacy [29, 35, 37]. A recent report indicated that DR3^+^ and DR4^+^ individuals may have a different course of disease, reflected, for example, by the difference in their response to teplizumab treatment [33]. Another recent study found that HLA type significantly influenced the effect of GAD65-alum therapy, with the best response to therapy observed in DR3^+^ DR4^−^ individuals [35].

Patients enrolled in the current study were not stratified by their HLA haplotype, and the treatment groups were not balanced for individuals positive for DR3 only, DR4 only, or DR3/DR4. Despite this limitation, the results of the subgroup analysis for these HLA types showed that patients who did not express HLA DR3 and who received IMCY-0098 doses B and C tended to have positive clinical outcomes. Favorable tendencies were also observed for patients who did express HLA DR4; however, owing to the study design, these two subgroups were nested: HLA DR3^−^ patients were all positive for DR4. To assess the difference between DR4-positive and DR3-negative patients, the analysis further explored the outcomes for DR3DR4 patients (DR4^+^ and DR3^+^): these patients did not show the favorable evolution that was observed on other DR4^+^ patients. Yet, given the small number of patients in this group (*n* = 5 for dose B + C), this difference between DR4^+^ and DR4^+^DR3^−^ patients must be interpreted with caution. As such, no firm conclusion regarding the differential impact of DR3 and DR4 on IMCY-0098 efficacy could be drawn. The difference in clinical outcomes between DR3 and DR4 subgroups may be due to disease heterogeneity [38]. Furthermore, the lack of clinical benefit observed for DR3 in this study may be due to the timeframe over which the study was conducted as the disease may progress slower in the DR3 (compared with the DR4) subgroup [39]. Another potential explanation may be observed in the immune response to treatment; both subgroups increased treatment-induced cytolytic CD4^+^ T cells, but the DR3 subgroup may have had a smaller increase than the DR4 subgroup, which was not considered in the KEM^®^ analysis. Finally, although in vitro characterization of IMCY-0098 indicated that it could bind similarly to DR3 and DR4 molecules [10], analysis of peptide-major histocompatibility complex (pMHC) stability showed that DR3 pMHC was less stable than DR4 pMHC (Imcyse data on file; data not shown). As pMHC stability influences the induction of the immune response [40], this may have in turn influenced the size of the cytolytic CD4^+^ T cell population in the DR3 subgroup.

In this exploratory analysis, clinical outcomes correlated with immune response in the IMCY-0098 dose B and C treatment groups, whereby improved clinical parameters were associated with an increase in cytolytic granzyme B^+^ CD4^+^ T cells and a decrease in pathogenic Perforin^+^ CD8^+^ T cells. Importantly, the increase in cytolytic CD4^+^ T cells appeared to be protective and did not deteriorate signs of disease. Recently, it has been reported that cytotoxic CD4 T cells are associated with T1D progression [41], but this was not observed in the current study. It is currently unknown if these cytotoxic CD4 T cells [41] are similar to the cytolytic CD4 T cells that increase upon treatment with IMCY-0098. Furthermore, although β cells have been reported to express MHC class II, there is no convincing evidence that they take up, process, and present auto-antigen-derived peptides [42]. If β cell MHC class II presentation was a predominant feature in T1D patients, we would have expected to observe disease exacerbation with IMCY-0098 treatment, as the MHC class II epitope IMCY-0098 contains (i.e., C20-A1) is known to be naturally processed from proinsulin [43]. Therefore, it is highly unlikely that cytolytic CD4^+^ T cells would kill β cells. These results support the probability that, at high doses, IMCY-0098 induces proinsulin-specific protective cytolytic CD4^+^ T cells and the subsequent elimination of pathogenic cytotoxic CD8^+^ T cells. This is consistent with the expected mechanism of action and the previous observations made in animal models [7].

The clinical data from the current trial have been published previously and showed that treatment with IMCY-0098 induced a reduction in some antibodies against T1D auto-antigens, suggesting a possible impact on specific T follicular helper cells by bystander killing [10]. The KEM^®^ platform analysis showed an IMCY-0098 dose-specific association with CD4 cells expressing IFN-γ and/or IL-17 (Additional File 1: Fig. S2). As Th17 cells are generally associated with autoimmune processes, the observed association could look counterintuitive; however, it has recently been described that different subtypes of Th17 cells exist [44]. This highlights the need for more precise characterization of major T cell populations involved in the T1D pathology process following treatment with IMCY-0098, such as single cell approaches that can be implemented in a longitudinal study follow-up.

The exploratory nature of this analysis and small sample size were limitations of this study. Increases in treatment-specific FOXP3^+^ Treg cells were not expected based on the postulated mechanism of action; therefore, the current study did not examine these cells. Further studies should examine these cells to evaluate the involvement of regulatory T cells and, to a broader extent, the different T-helper subtypes that could be impacted by IMCY-0098 treatment. Future studies should also test various treatment doses and schedules to demonstrate clinical proof-of-concept based on C-peptide secretion maintenance. While the results from a larger phase 2 trial are needed to support the findings presented here, the data-driven machine learning approach identified a number of hypotheses consistent with the clinical data, which will be incorporated into the experimental design of subsequent clinical studies. The preliminary results from this exploratory study are important to inform the design of further clinical studies of IMCY-0098 which will use selection of (or stratification for) HLA type and a more robust assessment of immune response focusing on the induction of cytolytic CD4^+^ T cells.

## Conclusions

This analysis showed that IMCY-0098 induces antigen-specific cytolytic CD4^+^ T cells and reduces the numbers of pathogenic CD8 + T cells in patients with T1D receiving the highest treatment dose. Due to the small study size and the exploratory nature of the machine learning-based analysis, this finding should be confirmed in larger studies, which will also help establish whether IMCY-0098 treatment efficacy is influenced by the patient’s HLA type. Furthermore, our data suggest that IMCY-0098 is a targeted, antigen-specific therapy; this mode of action may provide a better safety profile than non-specific immunosuppression although this deserves further study.

### Supplementary Information

Additional file 1: Supplementary Material. Fig. S1. Overview of main steps used to identify most relevant relationships between variables. Fig. S2. Immune response to IMCY-0098 treatment. Fig S3. Associations between clinical and immune response parameters. Fig. S4. Evolution of CD4+/Granzyme B+ CD107a cells in IMCY-0098 treated participants compared to placebo. Table S1. HLA haplotypes by cohort. Table S2. Formal concept analysis results. Table S3. Markers used for detection of cytolytic and pathogenic T cells combined with multiparameter flow cytometry.

## Data Availability

De-identified patient data can be provided to independent qualified researchers upon submission of a written application/research proposal that should be approved by the study sponsor.

## Abbreviations

APC: Antigen-presenting cell
AUC: Area under the curve
GAD65: Glutamic acid decarboxylase
HLA: Human leukocyte antigen
IFN: Interferon
IGRP: Islet-specific glucose-6-phosphatase catalytic subunit-related protein
IL: Interleukin
KEM^®^: Knowledge Extraction and Management
MMTT: Mixed meal tolerance test
PBMC: Peripheral blood mononuclear cells
T1D: Type 1 diabetes
