# Supplementary material for: First-in-human, double-blind, randomized phase 1b study of peptide immunotherapy IMCY-0098 in new-onset type 1 diabetes: an exploratory analysis of immune biomarkers
Source: BMC Med. 2024 Jun 21;22:259. doi: 10.1186/s12916-024-03476-y (PMC11191262; doi:10.1186/s12916-024-03476-y)
Supplement: Supplementary file 1 — Additional file 1: Supplementary Material. Fig. S1. Overview of main steps used to identify most relevant relationships between variables. Fig. S2. Immune response to IMCY-0098 treatment. Fig S3. Associations between clinical and immune response parameters. Fig. S4. Evolution of CD4+/Granzyme B+ CD107a cells in IMCY-0098 treated participants compared to placebo. Table S1. HLA haplotypes by cohort. Table S2. Formal concept analysis results. Table S3. Markers used for detection of cytolytic and pathogenic T cells combined with multiparameter flow cytometry. [file 12916_2024_3476_MOESM1_ESM.docx]

**First-in-human, double-blind, randomized phase 1b study of peptide immunotherapy IMCY-0098 in new-onset type 1 diabetes: an exploratory analysis of immune biomarkers**

**Supplementary material**

Jean Van Rampelbergh^1*†^, Peter Achenbach^2,3†^, Richard David Leslie^4†^, Martin Kindermans^5^, Frédéric Parmentier^5^, Vincent Carlier^1^, Nicolas Bovy^1^, Luc Vanderelst^1^, Marcelle Van Mechelen^1^, Pierre Vandepapelière^1†^, Christian Boitard^6,7†^

**Supplementary Figures**


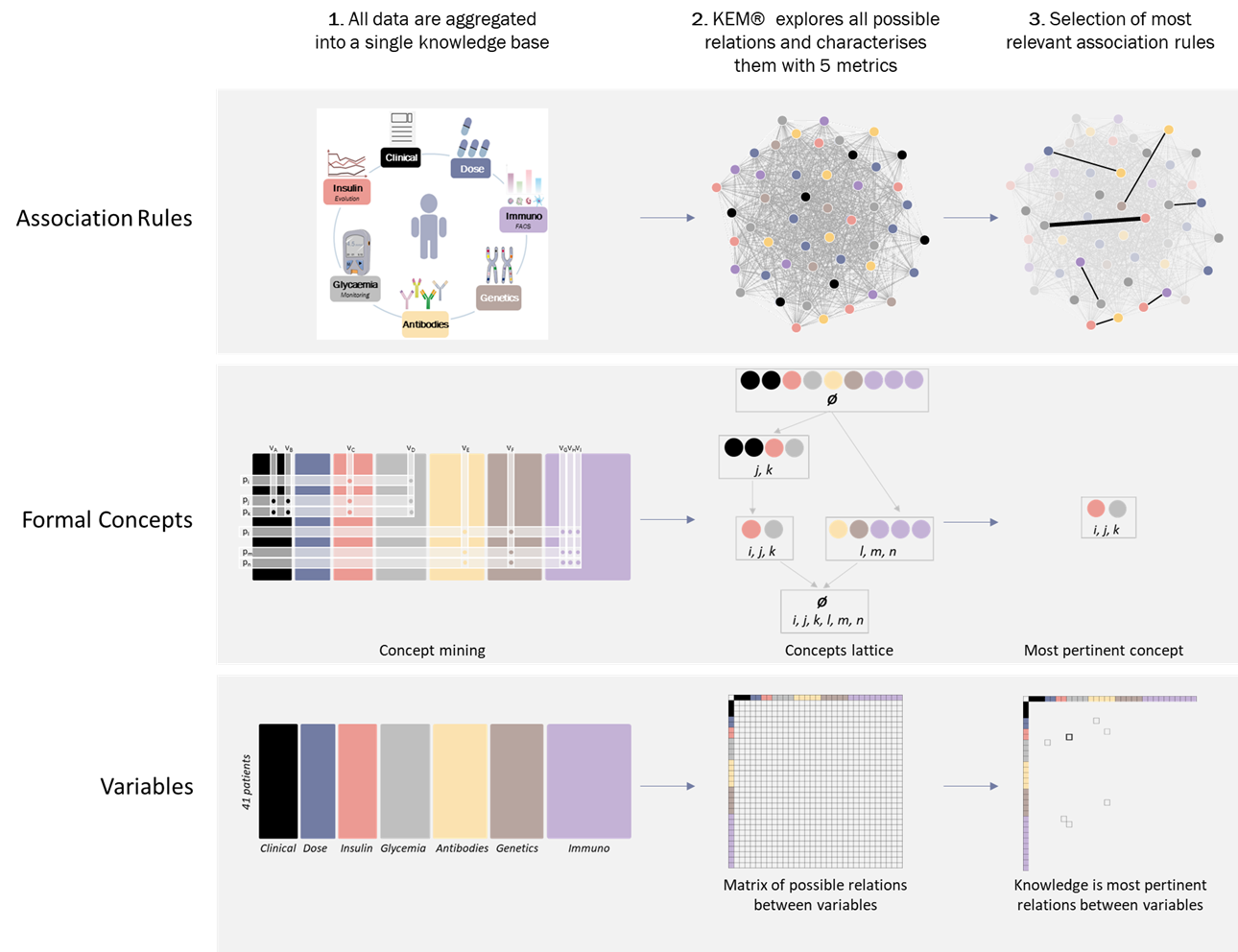


**Fig. S1.** Overview of main steps used to identify most relevant relationships between variables at three different levels: association rules, formal concepts, and variables. The association rules level illustrates the combinatorial complexity of the exploration phase. The formal concept level illustrates the theoretical framework organizing the generated association rules. The variables level illustrates how the data supports the generation of association rules and formal concepts as well as the overall explainability


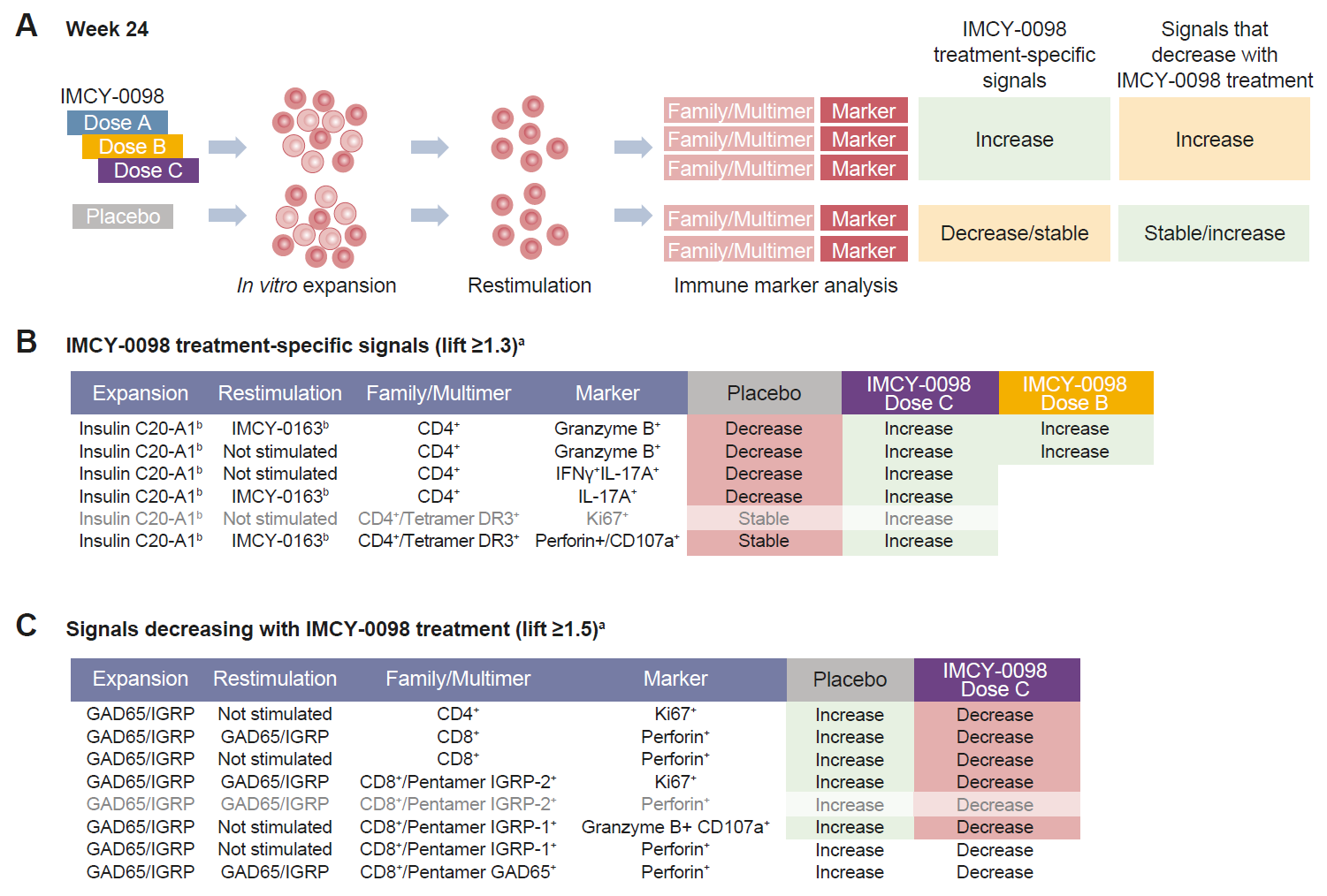


**Fig. S2.** Immune response to IMCY-0098 treatment: summary of immune parameters identified during formal concept analysis. (**A**) Peripheral blood mononuclear cells were analyzed after in vitro expansion and stimulation (or not) with indicated peptides. Presented are parameters that showed a positive (**B**) or negative (**C**) association with IMCY-0098 treatment and a converse association with placebo treatment. ^a^Associations were selected if they had confidence ≥0.75, support ≥4 (occurred in ≥4 patients) and had a *p*-value ≤0.05. Negative associations with IMCY-0098 were stronger than positive associations, leading to the use of different thresholds for the lift (the ratio probability of an outcome in a specific subgroup vs the probability of the outcome within all data sets). Parameters below the number of counts threshold in raw data are shown in light gray. ^b^Insulin C20-A1 (IMCY-0163) is the natural epitope contained in IMCY-0098. Dose A: 50 μg at Week 0 followed by 3 x 25 μg; dose B: 150 μg at Week 0 followed by 3 x 75 μg; dose C: 450 μg at Week 0 followed by 3 x 225 μg

**
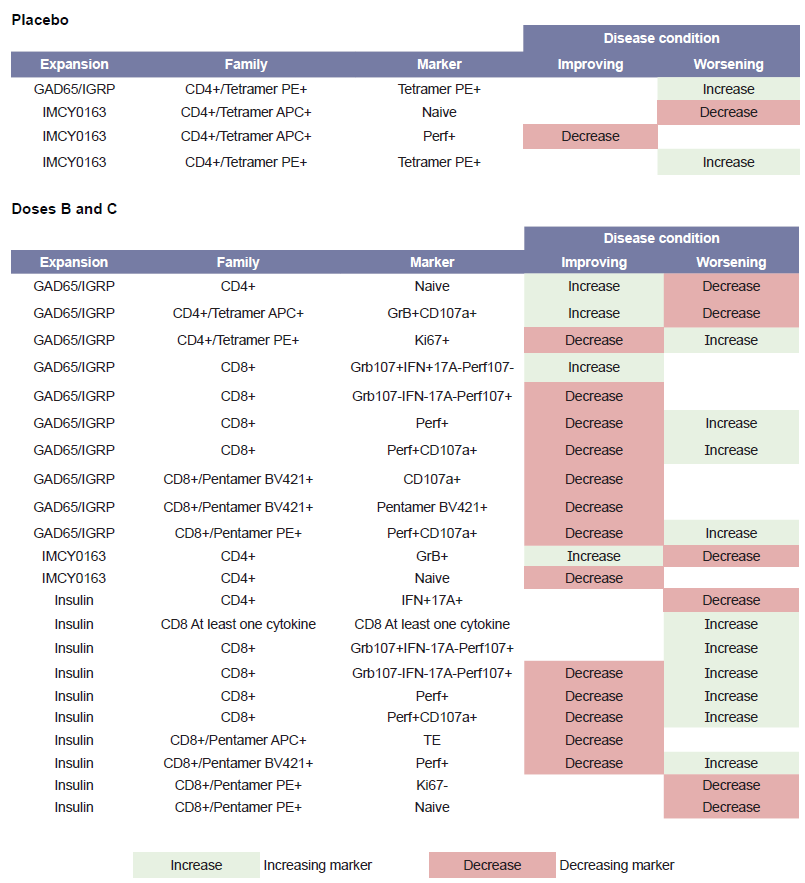
**

**Fig S3**. Associations between clinical and immune response parameters identified in formal concept analysis. Presented are parameters that showed a positive or negative association with placebo treatment or the ‘treated’ (Dose B and C) IMCY-0098 treatment groups.

**
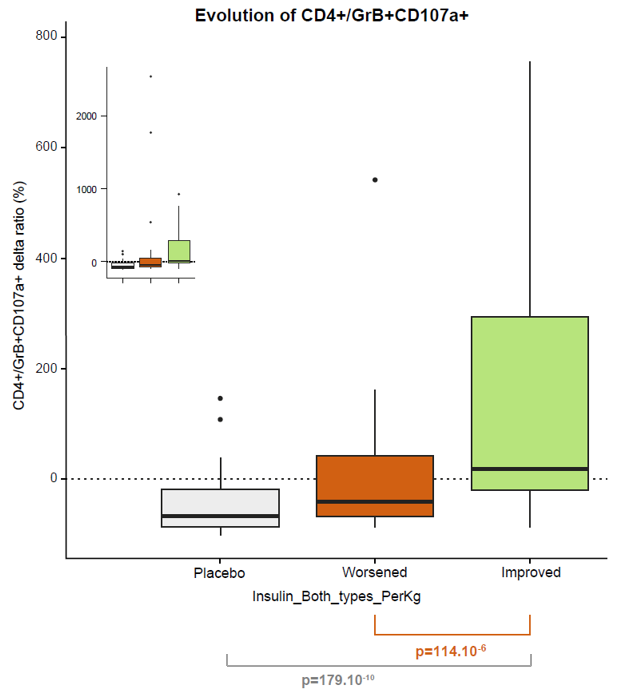
**

**Fig. S4.** Evolution of CD4^+^/Granzyme B^+^ CD107a cells in IMCY-0098 treated participants compared to placebo. Treated participants have been categorized based on their insulin use: improved was defined by a decrease in insulin use/kg and worsened was defined by an increase in the use of insulin/kg.

**Supplementary Tables**

**Table S1.** HLA haplotypes by cohort

| Haplotype, n (%) | **Placebo**  ***N*=10** | **Dose A**  ***N*=6** | **Dose B**  ***N*=9** | **Dose C**  ***N*=16** | **Total**  ***N*=41** |
| --- | --- | --- | --- | --- | --- |
| DR3 | 2 (20.0) | 2 (33.3) | 1 (11.1) | 5 (31.2) | 10 (24.4) |
| DR4 | 2 (20.0) | 3 (50.0) | 6 (66.6) | 8 (50.0) | 19 (46.3) |
| DR3/DR4 | 6 (60.0) | 1 (16.7) | 2 (22.2) | 3 (18.8) | 12 (29.3) |
| HLA-A2:01 | 8 (80.0) | 3 (50.0) | 5 (55.6) | 10 (62.5) | 26 (63.4) |

HLA, human leukocyte antigen

**Table S2.** Formal concept analysis results: associations between dose, candidate subgroups, clinical parameters and immune response at any visit

| **Treatment arm** | **Subgroup** | **Endpoint** | **Week** | **Endpoint increasing vs decreasing** | **Improvement vs worsening** |
| --- | --- | --- | --- | --- | --- |
| **IMCY-0098 Dose A** | AutoAb against GAD65 (+) | C-peptide/glucose | 18 | Decreased | Worsened |
|  | AutoAb against IA-2 (+) | C-peptide/glucose | 18 | Decreased | Worsened |
|  | AutoAb against Insulin (-) | C-peptide/glucose | 18 | Decreased | Worsened |
|  | AutoAb against ZnT8 (+) | C-peptide/glucose | 18 | Decreased | Worsened |
|  | C_max_ at baseline (low) | C-peptide/glucose | 18 | Decreased | Worsened |
|  | C-peptide/glucose at baseline (low) | C-peptide/glucose | 18 | Decreased | Worsened |
|  | Glucose at start at baseline (high) | C-peptide/glucose | 18 | Decreased | Worsened |
|  | Glycemia at baseline (high) | C-peptide/glucose | 18 | Decreased | Worsened |
|  | C_max_ at baseline (low) | C-peptide/glucose | 24 | Decreased | Worsened |
|  | C-peptide/glucose at baseline (low) | C-peptide/glucose | 24 | Decreased | Worsened |
|  | Glucose at start at baseline (high) | C-peptide/glucose | 24 | Decreased | Worsened |
|  | Glycemia at baseline (high) | C-peptide/glucose | 24 | Decreased | Worsened |
| **IMCY-0098 Dose C** | GAD at baseline (low) | C-peptide/glucose | 2 | Increased | Improved |
|  | HLA DR3^–^ | C-peptide/glucose | 4 | Increased | Improved |
|  | ZNT8 at baseline (low) | C-peptide/glucose | 4 | Increased | Improved |
|  | ZNT8 at baseline (low) | C-peptide/glucose | 6 | Increased | Improved |
|  | Age (high) | Normalized AUC (measured vs expected) | 12 | Decreased | Improved |
|  | HLA DR3^–^ | Normalized AUC (measured vs expected) | 12 | Decreased | Improved |
|  | HLA DR4^–^ | C-peptide/glucose | 12 | Decreased | Worsened |
|  | HLA DR4^–^ | Normalized AUC (measured vs expected) | 12 | Decreased | Worsened |
|  | HLA DR4^+^ | Normalized AUC (measured vs expected) | 12 | Decreased | Improved |
|  | Age (low) | C-peptide/glucose | 18 | Decreased | Worsened |
|  | BMI (low) | C-peptide/glucose | 18 | Decreased | Worsened |
|  | Age (high) | Normalized AUC (measured vs expected) | 24 | Decreased | Improved |
|  | HLA DR3^–^ | Normalized AUC (measured vs expected) | 24 | Decreased | Improved |
|  | HLA DR4^+^ | Normalized AUC (measured vs expected) | 24 | Decreased | Improved |
|  | Time to diagnosis (low) | Normalized AUC (measured vs expected) | 24 | Decreased | Improved |
| **Placebo** | AUC at baseline (high) | C-peptide/glucose | 2 | Decreased | Worsened |
|  | BMI (high) | C-peptide/glucose | 2 | Decreased | Worsened |
|  | HLA DR3^+^ | C-peptide/glucose | 2 | Decreased | Worsened |
|  | Gender (female) | C-peptide/glucose | 2 | Decreased | Worsened |
|  | AutoAb against Insulin (+) | C-peptide/glucose | 4 | Decreased | Worsened |
|  | AutoAb against GAD65 (-) | C-peptide/glucose | 6 | Increased | Improved |
|  | C-peptide/glucose at baseline (low) | C-peptide/glucose | 6 | Increased | Improved |
|  | Fasting C-peptide at baseline (low) | C-peptide/glucose | 12 | Increased | Improved |
|  | Fasting C-peptide at baseline (low) | Normalized AUC (measured vs expected) | 12 | Decreased | Improved |
|  | AutoAb against GAD65 (-) | C-peptide/glucose | 18 | Increased | Improved |
|  | C-peptide/glucose at baseline (low) | C-peptide/glucose | 18 | Increased | Improved |
|  | Fasting C-peptide at baseline (low) | C-peptide/glucose | 18 | Increased | Improved |
|  | GAD at baseline (low) | C-peptide/glucose | 18 | Increased | Improved |
|  | HLA DR4^+^ | C-peptide/glucose | 18 | Increased | Improved |
|  | Gender (male) | C-peptide/glucose | 18 | Increased | Improved |

The analysis focused on two clinical endpoints: C-peptide/glucose ratio and the difference between the measured and the expected normalized AUC C-peptide from MMTT. Associations were selected if they had confidence ≥0.75, support ≥4 (occurred in ≥4 patients), and it had a *p*-value ≤0.05. For each parameter, categories of low, medium, and high were assigned to values in the lowest, middle, and highest tertiles, respectively, of all patient values. Dose A: 50 μg at Week 0 followed by 3 x 25 μg; dose B: 150 μg at Week 0 followed by 3 x 75 μg; dose C: 450 μg at Week 0 followed by 3 x 225 μg

AUC, area under the curve; AutoAb, auto-antibody; BMI, body mass index; C_max_, maximum concentration; GAD, glutamic acid decarboxylase; HLA DR, human leukocyte antigen DR haplotype; IGRP, islet-specific glucose-6-phosphatase catalytic subunit-related protein; MMTT, mixed meal tolerance test

**Table S3.** Markers used for detection of cytolytic and pathogenic T cells combined with multiparameter flow cytometry

| **Response** | **T-cell population** | **MHC-I/II multimers** | **Stimulatory peptides** |
| --- | --- | --- | --- |
| **Immune regulation** | Cytolytic CD4^+^ T cells | MHC-II (DRB1*03:01 and DRB1*04:01) tetramers | Proinsulin C20-A1 (natural epitope contained in IMCY-0098) |
| **Effector response** | Pathogenic (islet-specific) CD4^+^ T cells | MHC-II (DRB1*03:01 and DRB1*04:01) tetramers | GAD65 epitope (Peptivator® human GAD65, Miltenyi, Bergisch Gladbach, Germany); IGRP major epitopes (Lifetein, Somerset NJ, USA) |
|  | Pathogenic (islet-specific) CD8^+^ T cells | MHC-I (HLA-A*02:01) pentamers | Insulin epitopes (Peptivator® human insulin, Miltenyi, Bergisch Gladbach, Germany), GAD65 or IGRP epitopes (see above) |
| **Multiparameter FACS markers and antibody source** | | | |
| **Marker** | **Dye** | **Species, type** | **Source** |
| **Viability** |  |  |  |
| Live/dead assay | N/A | N/A | ThermoFisher Scientific |
| Phenotype |  |  |  |
| CD3 | BUV737 | Mouse IgG1 | BD Biosciences |
| CD4 | BV711 | Mouse IgG1 | BD Biosciences |
| CD8 | BV786 | Mouse IgG1 | BD Biosciences |
| **T cell subsets** |  |  |  |
| CCR7 | PE-Cy7 | Mouse IgG1 | BioLegend |
| CD45RA | APC-Cy7 | Mouse IgG1 | BioLegend |
| **Proliferation** |  |  |  |
| Ki67 | PE-eFluor610 | Mouse IgG1 | eBioscience |
| **Function** |  |  |  |
| IFN-γ | FITC | Mouse IgG1 | BioLegend |
| IL-17A | BV605 | Mouse IgG1 | BioLegend |
| CD107a | BUV395 | Mouse IgG1 | BD Biosciences |
| Granzyme B | Alexa Fluor 700 | Mouse IgG1 | BD Biosciences |
| Perforin | PerCP-Cy5.5 | Mouse IgG1 | BioLegend |
| **Multimer loading** | | | |
| **Multimer** | **Dye** | **Antigen source** | **Epitope sequence** |
| DRA1*01:01/DRB1*0301 | PE | Proinsulin C20-A1 | SLQPLALEGSLQKRG |
| DRA1*01:01/DRB1*0401 | APC | Proinsulin C20-A1(K/S) | SLQPLALEGSLQSRG |
| DRA1*01:01/DRB1*0301 | PE | GAD65 339-352 | TVYGAFDPLLAVAD |
| DRA1*01:01/DRB1*0401 | APC | GAD65 274-286 | IAFTSEHSHFSLK |
| A*02:01 | PE | Preproinsulin L15-L24 | ALWGPDPAAA |
| A*02:01 | APC | Preproinsulin L6-L14 | RLLPLLALL |
| A*02:01 | BV421 | Proinsulin B10-B18 | HLVEALYLV |
| A*02:01 | PE | GAD65 114-122 | VMNILLQYV |
| A*02:01 | APC | IGRP 265-273 | VLFGLGFAI |
| A*02:01 | BV421 | IGRP 228-236 | LNIDLLWSV |

CD, cluster of differentiation; FACS, fluorescence-activated cell sorting; GAD, glutamic acid decarboxylase; IFN-γ, interferon gamma; IGRP, islet-specific glucose-6-phosphatase catalytic subunit-related protein; HLA, human leukocyte antigen; IL, interleukin; MHC, major histocompatibility complex; N/A, not applicable
